# A Deep Learning-Based Method for Automatic Assessment of Stomatal Index in Wheat Microscopic Images of Leaf Epidermis

**DOI:** 10.3389/fpls.2021.716784

**Published:** 2021-09-03

**Authors:** Chuancheng Zhu, Yusong Hu, Hude Mao, Shumin Li, Fangfang Li, Congyuan Zhao, Lin Luo, Weizhen Liu, Xiaohui Yuan

**Affiliations:** ^1^School of Computer and Artificial Intelligence, Wuhan University of Technology, Wuhan, China; ^2^State Key Laboratory of Crop Stress Biology for Arid Areas, College of Plant Protection, Northwest A&F University, Shaanxi, China; ^3^Chongqing Research Institute, Wuhan University of Technology, Chongqing, China; ^4^Engineering Research Centre of Chinese Ministry of Education for Edible and Medicinal Fungi, Jilin Agricultural University, Changchun, China; ^5^Jiaxing Key Laboratory for New Germplasm Breeding of Economic Mycology, Jiaxing, China

**Keywords:** stomatal index, cell counting, stomata detection, convolutional network, transfer learning

## Abstract

The stomatal index of the leaf is the ratio of the number of stomata to the total number of stomata and epidermal cells. Comparing with the stomatal density, the stomatal index is relatively constant in environmental conditions and the age of the leaf and, therefore, of diagnostic characteristics for a given genotype or species. Traditional assessment methods involve manual counting of the number of stomata and epidermal cells in microphotographs, which is labor-intensive and time-consuming. Although several automatic measurement algorithms of stomatal density have been proposed, no stomatal index pipelines are currently available. The main aim of this research is to develop an automated stomatal index measurement pipeline. The proposed method employed Faster regions with convolutional neural networks (R-CNN) and U-Net and image-processing techniques to count stomata and epidermal cells, and subsequently calculate the stomatal index. To improve the labeling speed, a semi-automatic strategy was employed for epidermal cell annotation in each micrograph. Benchmarking the pipeline on 1,000 microscopic images of leaf epidermis in the wheat dataset (*Triticum aestivum* L.), the average counting accuracies of 98.03 and 95.03% for stomata and epidermal cells, respectively, and the final measurement accuracy of the stomatal index of 95.35% was achieved. *R*^2^ values between automatic and manual measurement of stomata, epidermal cells, and stomatal index were 0.995, 0.983, and 0.895, respectively. The average running time (ART) for the entire pipeline could be as short as 0.32 s per microphotograph. The proposed pipeline also achieved a good transferability on the other families of the plant using transfer learning, with the mean counting accuracies of 94.36 and 91.13% for stomata and epidermal cells and the stomatal index accuracy of 89.38% in seven families of the plant. The pipeline is an automatic, rapid, and accurate tool for the stomatal index measurement, enabling high-throughput phenotyping, and facilitating further understanding of the stomatal and epidermal development for the plant physiology community. To the best of our knowledge, this is the first deep learning-based microphotograph analysis pipeline for stomatal index assessment.

## Introduction

Stomata are formed by pairs of specialized epidermal guard cells, which are the main pathways for gas exchange in the essential physiological processes of leaf plants, such as carbon assimilation, respiration, and transpiration (Kim et al., 2010). The counting and measuring of stomata in microscopic images of leaf epidermis have been one of the most typical plant biological activities (Willmer and Fricker, 1996). The stomatal density and size are good indicators that reflect the response of plants to abiotic stresses in the environment and permit quantitative estimation of the stomatal gas exchange parameters (Sack and Buckley, 2016). However, these traits will alter with the growth of plants or the environment that cannot be used to reveal the stomata initiation and epidermal development across plant genotypes or species. The stomatal index, estimated as the number of stomata per number of epidermal cells plus stomata, is relatively constant during plant growth (Salisbury, 1928). It is of greater significance in studying the epidermal development process in plant physiology and their genetic basis in plant breeding for productivity (Royer, 2001; Sack and Buckley, 2016).

The microscopic images of plant leaf epidermis contain two types of cells, namely, the tightly arranged epidermal cells and the guard cells. Stomata in the leaf epidermis are bounded by the bean- or dumbbell-shaped guard cells with fixed shapes, and in some species but not all, they are surrounded by one-to-many subsidiary cells (Boetsch et al., 1996). At present, various image analysis tools have been developed for detecting, counting (Aono et al., 2019; Fetter et al., 2019), and measuring stomatal aperture (Omasa and Onoe, 1984; Li et al., 2019) as well as assessing stomatal density (Vialet-Chabrand and Brendel, 2014). However, to the best of our knowledge, there is no pipeline designed for the stomatal index measurement, possibly due to the difficulty in epidermal cell detection. For that reason, this study aims to develop a pipeline for automatically measuring stomatal index by simultaneously counting epidermal cells and stomata from microscopic images of plant epidermis.

So far, many image processing-based stomata analysis tools have been proposed for a diversity of plant species. For tomato, Sanyal et al. (2008) isolated the stomata using a watershed algorithm, eliminated noise using morphological operations, and extracted the stomatal edges using the Sobel operator to measure the morphological features of the stomata (e.g., area, center of gravity, and compactness). As an edge-based method, its performance is insufficient when the edge of the stoma is discontinuous or has considerable noise. Laga et al. (2014) proposed a fully automatic tool for phenotyping the length and width of stomata openings and the size of guard cells in wheat. But this tool relied on a template-matching technique to detect stomata, which reduced its versatility in the presence of considerable variability in the stomata shapes. Another automatic method for stomata detection and counting used morphological operations (Da Silva Oliveira et al., 2014). This method required relatively high image quality and was not robust to images containing impurities. These disadvantages of image processing-based methods have led to the adoption of more advanced computer vision techniques. Recently, deep learning techniques, especially convolutional neural networks (CNNs), have emerged as powerful methods for automatically training the feature detector with the classifier. They made remarkable achievements in a range of object detection tasks. Stomata recognition is not an exception. General one-stage object detection algorithms, single shot multiBox detector (SSD, Sakoda et al., 2019) and you only look once (YOLO, Casado-García et al., 2020), and two-stage object detection algorithm, real-time object detection with Faster R-CNN (Li et al., 2019) and mask region-based CNN (Mask R-CNN, Bheemanahalli et al., 2021) built accurate stomata detection models for many plant species such as rice, soybean, wheat, barley, or sorghum. This study selected the Faster R-CNN for detecting and counting stomata by considering the speed-accuracy trade-off of the model.

Identifying and counting epidermal cells in leaf images are vital for developing the stomatal index measurement algorithm. Unlike stomata with fixed shapes and distinct morphological features, epidermal cells exhibit great diversities in size, shape, and clustering in different plant species. The epidermal cells of the wheat leaf are long, thin, transparent, and tightly touching with one another. Although we attempted multiple generic object detection algorithms, none of them achieved satisfying performance in recognizing epidermal cells. U-Net, a deep-learning model designed for frequently occurring quantification tasks such as cell detection and semantic segmentation in biomedical image data (Ronneberger et al., 2015), may be a suitable solution. Modified and extended from a fully convolutional network (FCN), U-Net used excessive elastic deformations for data augmentation and trained on a diverse set of data, allowing adaption to new tasks with a small number of annotated images. Recently, some animal cell segmentation studies, such as bladder cancer cell segmentation in phase-contrast microscopy images (Hu et al., 2019) and nuclei segmentation (Zeng et al., 2019) were based on the U-Net structure. Some studies of smart farming showed promising performance using U-Net, such as segmentation of cucumber leaf disease (Lin et al., 2019) and field study of wheat yellow rust monitoring (Su et al., 2020). A weight loss in U-Net was designed for isolating background labels between touching cells, which is suitable for the detection task of epidermal cells. Unfortunately, there is currently no example of applying U-Net to plant cell segmentation to the best of our knowledge.

In this study, we developed an automatic image analysis pipeline to assess the stomatal index from microscopic images of the leaf epidermis. Faster R-CNN was deployed to count the stomata number. U-Net was utilized to extract the epidermal cell network. After a series of morphological image post-processing, the number of epidermal cells was calculated by counting the number of connected domains from the epidermal cell network. Finally, the stomatal index of the current microscopic image was calculated by dividing the stomata number by the total number of stomata and epidermal cells.

## Materials and Methods

### Image Acquisition

#### Wheat Dataset

**Figure 1:**
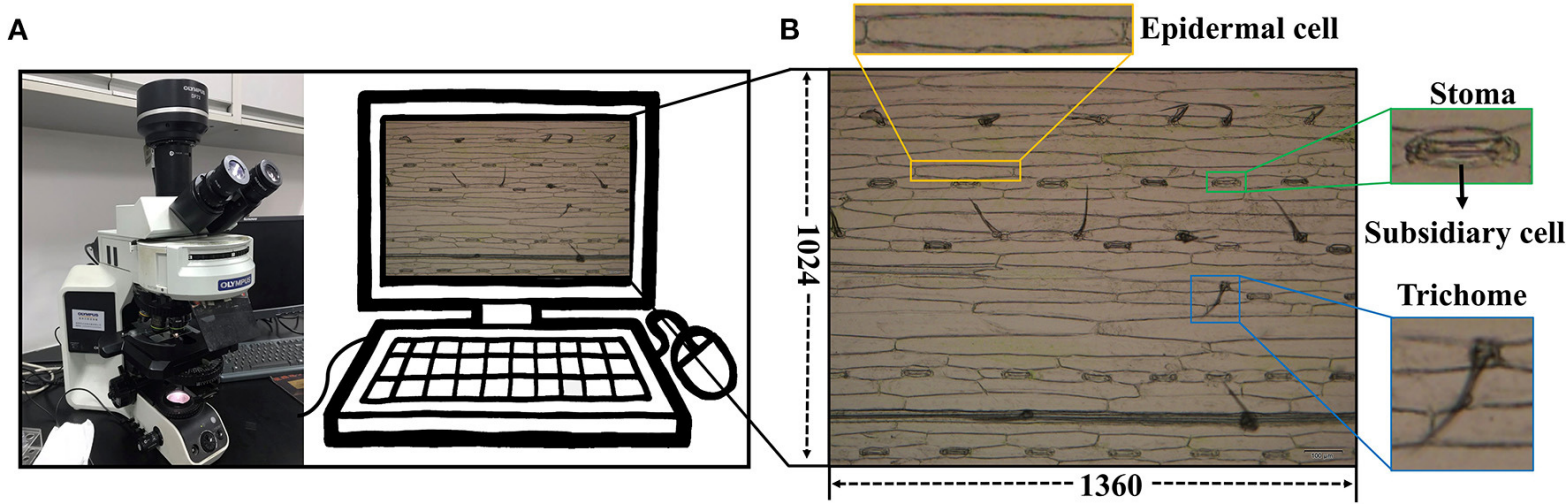
Data collection platform. **(A)** Olympus DP72 industrial microscope. **(B)** Microscope image of wheat leaf epidermis.

A total of 1,000 microscopic images was collected from the leaf abaxial epidermis of fully expanded leaves derived from 100 wheat varieties (*Triticum aestivum* L., Supplementary Table 1). Seedlings were grown in a growth chamber at 14°C with 16 h of light and 8 h of darkness. At the two-leaf stage, the fully expanded second leaves were cut from the plants. We stuck the abaxial surface of the collected wheat leaves on tapes and scraped off the epidermis and mesophyll cells at the adaxial surface of the leaves with a sharp scalpel, leaving only the colorless and transparent abaxial epidermal cells attached to the tape. The tape with intact and clean abaxial epidermal cells and stomata was stuck onto a clean slide and then imaged the leaf areas on either side of the primary veins at × 10 magnification using the Olympus DP72 microscope camera (Figure 1A). Five micrographs were taken for each specimen, and a total of 15 micrographs were collected for three specimens of each variety. They were stored in JPEG format with a resolution of 1,360 × 1,024 (Figure 1B). A total of 500 images, were randomly selected and cropped to 680 × 512 and reshaped to 1,360 × 1,024 using cubic interpolation to obtain 500 images at × 20 magnification. In the end, 500 images at × 20 magnification and the remaining 500 images at × 10 magnification were combined as the wheat dataset for this study.

#### Cuticle Dataset

To verify whether the pipeline can be applied to other species, 156 microscopic images of plant cuticles derived from 31 plant families (Supplementary Table 2) were downloaded from the cuticle database (https://cuticledb.eesi.psu.edu/, Barclay et al., 2007). As shown in Supplementary Figure 1, the morphologies of stomata and epidermal cells of these 31 plant families are pretty diverse. These micrographs were obtained *via* imaging the specimens of stained leaf tissues (Barclay et al., 2007; Fetter et al., 2019).

### Automatic Pipeline for Stomatal Index Measurement

**Figure 2:**
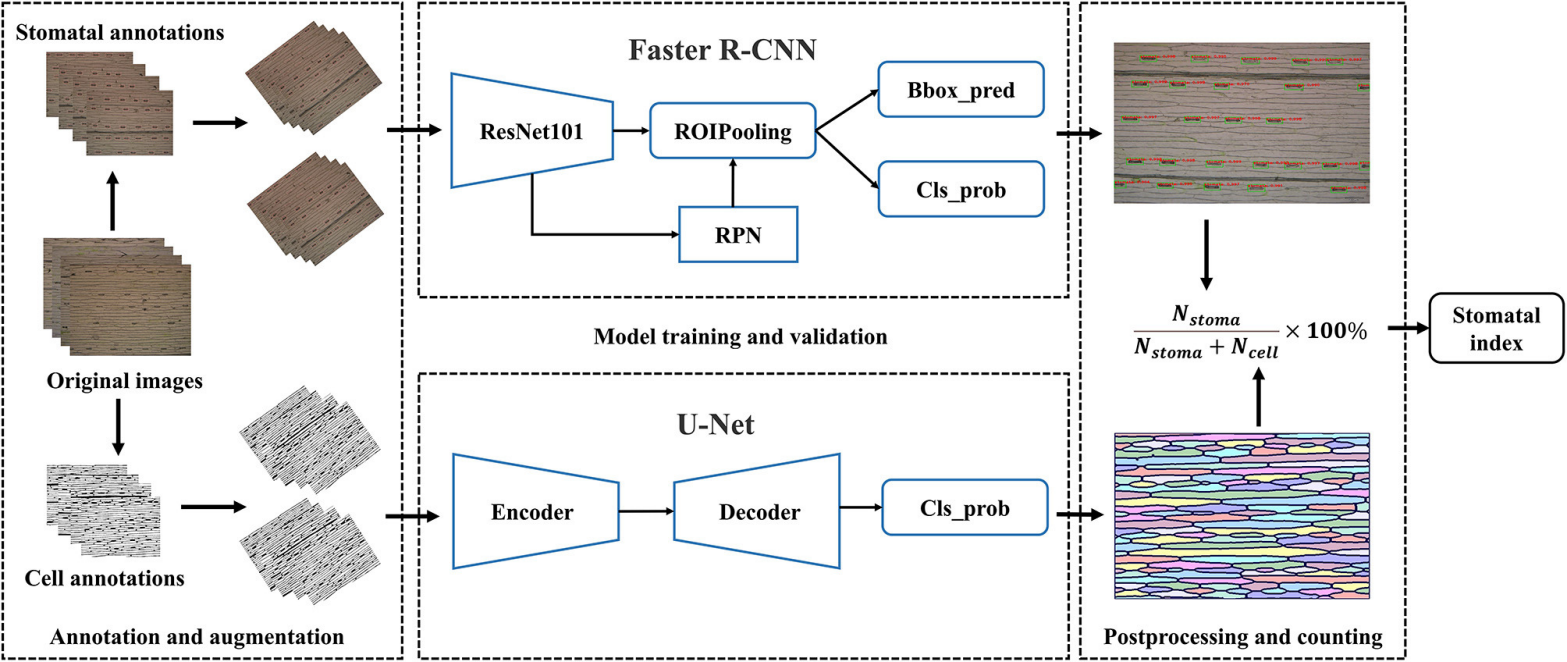
A flow chart of the proposed pipeline of the stomatal index measurement.

We developed a fully automatic solution for stomatal index measurement that mainly consisted of two parts, namely, stomata and epidermal cell counting (Figure 2). The details are described as follows: At first, the microscopic images of crop leaves were annotated and augmented to build the dataset. The Faster R-CNN was used to identify the stomata and counting in a given microscopic image. The U-Net was employed to segment the epidermal cells as connected domains. Several image-processing techniques were applied to refine the segmentation results of U-Net based on cell morphological features of epidermal cells. The number of epidermal cells in a given microscopic image was measured by counting the number of high-quality connected domains. Subsidiary cells associated with the guard cells were present in wheat and many (but not all) plant families of the cuticle dataset (Figure 1B and Supplementary Figure 1). It should be noted that they were not counted as epidermal cells in the study. Finally, the stomatal index was calculated by the following formula.

(1) $$\begin{array}{l} stomatal\;index=\frac{stomata\;number}{\left(stomata\;number\;+epidermal\;cell\;number\right)}\times 100\% \end{array}$$

### Image Annotation

**Figure 3:**
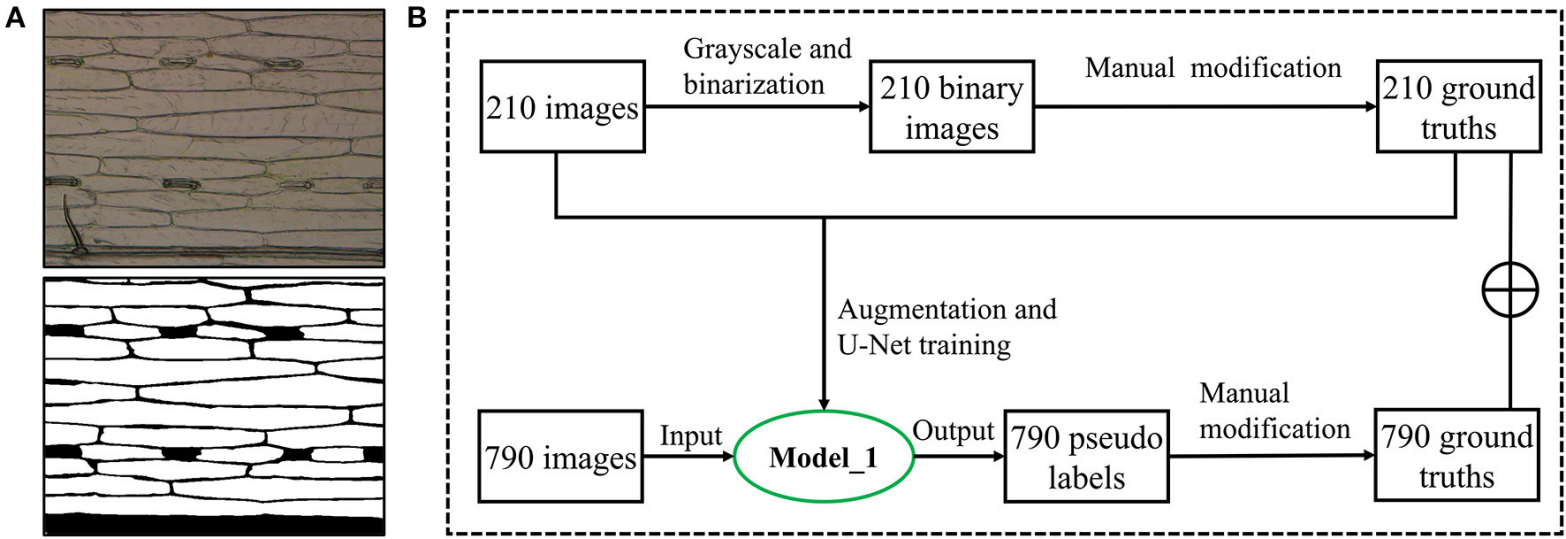
Epidermal cell annotation. **(A)** Original image of wheat leaf epidermis microscope (upper) and its annotation image (lower). **(B)** Semiautomatic annotation process using an initial segmentation model (Model_1).

Stomata in the 1,000 microscopic images were manually annotated by three experts using the Colabeler (AI labeling tool, http://www.colabeler.com). For each annotated image, an additional extensible markup language (XML) file with the same name as the original image file was exported in Pascal VOC format of object detection (Everingham et al., 2010). Each stoma was marked by the smallest circumscribed rectangle {x_min_, y_min_, x_max_, y_max_} to determine its relative position on the image. All the complete stomata or stomata with more than half of the length at the edge of the image were marked. For epidermal cell annotation, we utilized the semi-automatic strategy considering a large number of epidermal cells in each microscopic image. There were two types of annotations for each epidermal cell segmentation experiment: black and white. We used black (RGB: 0, 0, 0) to label the cell wall or leaf vein and white (RGB: 255, 255, 255) to label the intracellular regions. In addition, we ignored trichomes by marking them as white to eliminate their interferences for epidermal cell segmentation. Subsidiary cells were labeled in black since they were not counted as epidermal cells (Figure 3A). Usually, it is very time-consuming and laborious to obtain the ground truth of the semantic segmentation task. A semiautomatic annotation method was used to improve the efficiency of cellular annotation. The whole annotation process was shown in Figure 3B. In brief, 210 images were grayscaled, binarized, and manually annotated using Microsoft Paint 3D for Windows. These annotated images trained the U-Net with image augmentation for 200 epochs to obtain an initial segmentation model (Model_1). The remaining 790 images in the dataset were fitted into Model_1 to generate corresponding pseudo labels. After manual modification to the ground truths, all the 1,000 microscopic images were annotated. The proposed semiautomatic annotation method was much more efficient than the manual method. It took about 10 min to generate an annotation with manual correction, while fully manual annotation cost about 1 h per image.

### Deep Learning-Based Algorithms

#### Stomata Detection by Faster R-CNN

ResNet101 (He et al., 2016) was implemented as the backbone of Faster R-CNN to extract feature maps. In this network, the region proposal network (RPN) was used to scan the backbone feature map, which effectively reused the extracted features and avoided repeated calculation of region of interest (ROI). The region proposal generated by RPN was combined with the feature map obtained in the last layer of Resnet101 to generate a fixed size proposal feature map using ROIPooling, and prepare for the following full connection operation target classification and location regression. After that, softmax was used for object classification. In addition, the smooth L1 loss was adopted to complete the bounding box (bbox) regression operation and obtain the exact position of the object. The loss function is as follows:

(2) $$\begin{array}{l} L\left(\left\{p_{i}\right\},\left\{t_{i}\right\}\right)=\frac{1}{N_{cls}}\underset{i}{\sum }L_{cls}\left(p_{i},p_{i}^{*}\right)+\lambda \frac{1}{N_{reg}}\underset{i}{\sum }p_{i}^{*}L_{reg}\left(t_{i},t_{i}^{*}\right) \end{array}$$

(3) $$\begin{array}{l} smooth_{L1}\left(x\right)=\{\begin{array}{cc} 0.5x^{2} & if|x|<1\\ |x|-0.5 & otherwise \end{array} \end{array}$$

where *L*_*cls*_ is the Softmax Loss, $L_{reg}\left(t_{i},t_{i}^{*}\right)=smooth_{L1}\left(t_{i}-t_{i}^{*}\right)$ defined in Formula (3) and λ =10.

We trained the Faster R-CNN model with a specific configuration. The general idea is to use mini-batch gradient descent. The batch size was set to 16, and the input image size was adjusted to 800 × 600, considering the limited graphics processing unit (GPU) memory. The initial learning rate was 0.0005 and attenuated to 0.00005 during 20 epochs using the cosine annealing algorithm (Loshchilov and Hutter, 2016). Considering the relatively small proportion of stomata in the image, we set the anchor scale to 4, 8, 16, and anchor ratios to 0.5, 1, and 2. The loss weight of the RPN class, RPN bbox, Faster R-CNN class, and Faster R-CNN bbox were the same. Details of other parameters can be obtained from the literature (Ren et al., 2015). The backbone of the Faster R-CNN was initialized by the ResNet101 pretrained model of ImageNet (Deng et al., 2009), and the other parts were initialized randomly by a normal distribution.

#### Epidermal Cell Segmentation by U-Net

We applied the U-Net, a semantic segmentation network specially designed for biomedical images (Ronneberger et al., 2015), for segmenting leaf epidermal cells. Since semantic segmentation belongs to the classification of each pixel, it is very sensitive to light. To better extract, the cellular network, each channel of the training image was standardized before training using formula (4). The resolution of the image was adjusted to 512 × 512 for training the U-Net model, which is a compromise between obtaining as many image details as possible and GPU memory limit.

(4) $$\begin{array}{l} \hat{I}=\frac{I-\mu }{adjusted\text{\_}stddev}\;,adjusted\text{\_}stddev=max\left(\sigma ,\frac{1.0}{\sqrt{N}}\right) \end{array}$$

where μ is the mean value of all image pixels in the dataset, *I* is an image matrix, σ is the SD of all image pixels in the dataset, and *N* is the number of pixels in *I*, Î is the standardized image matrix.

Parameters for the training model were as follows: the initial learning rate was 0.0001 (Smith, 2017); it was decayed to 0.00001 by the cosine annealing algorithm (Loshchilov and Hutter, 2016) during 200 epochs; the batch size was set as 8. Kaiming initialization method (He et al., 2015) was used for the initialization of the weights. The loss function was binary cross-entropy, as shown in Formula (5).

(5) $$\begin{array}{l} H_{p}\left(q\right)=-\frac{1}{N}\overset{N}{\underset{i=1}{\sum }}y_{i}\cdot \log\left(p\left(y_{i}\right)\right)+\left(1-y_{i}\right)\cdot \log\left(1-p\left(y_{i}\right)\right) \end{array}$$

#### Stomatal Index Measurement

Both the Faster R-CNN-based stomata detection algorithm and U-Net-based epidermal cell segmentation algorithm did not export the number of stomata and epidermal cells. Before measuring the stomatal index in each microscopic image, we need to count stomata and epidermal cells. The stomata detection model returned a series of five-dimensional vectors for each microscope image, given by {score, xmin, ymin, xmax, ymax}. The score in the vector represents the confidence of each bbox, and the following four parameters represent the position of the bbox on the image. To avoid counting low-probability stomata within the noise, the bbox with a score >0.9 was counted as a stoma.

Epidermal cell segmentation by U-Net generated a corresponding cell network image for each leaf microscopic image. We regarded each connected domain as an epidermal cell and counted the number of connected domains in each image as the epidermal cell number. Before counting, bilateral filtering (Tomasi and Manduchi, 1998) was used to remove noise from the prediction, binarization, and morphological opening operations (first erosion and then dilation) were performed to connect breaks of some cell walls. The incomplete connected domains were filtered out if their pixel numbers were <1/10 of the average pixel number of all connected domains in the image. In the end, the stomatal index of each microscopic image was calculated as the ratio of stomata number to the total number of stomata and epidermal cells, as shown in the Formula (1).

#### Performance Evaluations

We evaluated the performance of the stomata detection algorithm using average precision (AP), which is defined as the area under an interpolated precision-recall curve. The AP was computed as follows:

(6) $$\begin{array}{l} AP=\underset{0}{\overset{1}{\int }}P\left(R\right)dR \end{array}$$

where precision is $P=\frac{N_{TP}}{N_{TP}+N_{FP}}$ and recall is $R=\frac{N_{TP}}{N_{TP}+\;N_{FN}}$. *N*_*TP*_ is the true positive, *N*_*TN*_ is the true negative, *N*_*FP*_ is the false positive, and *N*_*FN*_ is the false negative.

The performance of the epidermal cell segmentation algorithm was assessed using the Dice Coefficient (DC) (Formula 7), which compares the overlap rate of segmentation results of the models with the manual annotation.

(7) $$\begin{array}{l} Dice\;coefficient=\frac{2N_{TP}}{2N_{TP}+N_{FP}+N_{FN}} \end{array}$$

where *N*_*TP*_, *N*_*FP*_, and *N*_*FN*_ represent the true positives, false positives, and false negatives of pixel numbers, respectively.

As shown in Formula 8, counting accuracy was defined to evaluate the stomata and epidermal cell counting pipeline performance. We assumed the manual counts by experts containing true positives and the automatic counts by the image analysis pipeline containing true positives and false positives. Therefore, counting error could be obtained by subtracting the automatic results from the manual results, and the counting accuracies for stomata and epidermal cells were defined as:

(8) $$\begin{array}{l} Counting\;accurary=1-\frac{abs\left(Automatic\;Count-Manual\;Count\right)}{Manual\;Count} \end{array}$$

To detect the overcounting and undercounting errors, we defined counting precision as:

(9) $$\begin{array}{l} Counting\;precision=log\left(\frac{Manual\;Count}{Automatic\;Count}\right) \end{array}$$

The negative values of counting precision indicated the overcounting errors, and the positive values indicated undercounting errors. The counting precision was undefined when the manual count or the automatic count is zero. Simple linear regression was also applied to explore the relationship between manual and automated counting of stomata and epidermal cells.

To explore the accuracy and precision of stomatal index measurement, we calculated the stomatal index using the manual results of stomata and epidermal cells and their automatic results. Equations were defined as:

(10) $$\begin{array}{l} Stomatal\;index\;accuracy\;=\\ 1-\frac{abs\left(Automatic\;result-Manual\;result\right)}{Manual\;result}\; \end{array}$$

(11) $$\begin{array}{l} Stomatal\;index\;precision=log\left(\frac{Manual\;result}{Automatic\;result}\right) \end{array}$$

We assessed the measurement speed of the stomatal index on the central processing unit (CPU) and GPU (1080Ti) by calculating the average running time (ART) for each image. The measurement time of the stomatal index is equal to the sum of stomata counting time and cell counting time.

(12) $$\begin{array}{l} ART=\frac{T_{N\text{\_}stoma}+T_{N\text{\_}cell}+T_{N\text{\_}SI}}{N} \end{array}$$

where *N* represents the number of images, *T*_*N*_*stoma*_, *T*_*N*_*cell*_, and *T*_*N*_*SI*_ represent the total running time for counting stomata and cells and stomatal index formula calculation for N images, respectively.

### Statistical Analysis

R version 3.6 (R Core Team, 2020) was used to perform simple linear regressions (*y* = *x*) for assessing the linear relationship between manual counting by experts and automatic counting by the proposed pipeline. The equations of coefficient of determination (*R*^2^) and root mean square error (RMSE) for the simple linear regression are as follows:

(13) $$\begin{array}{l} R^{2}=1-\frac{\sum_{i}{\left(x_{i}-y_{i}\right)}^{2}}{\sum_{i}{\left(x_{i}-ȳ\right)}^{2}} \end{array}$$

(14) $$\begin{array}{l} RMSE=\sqrt{\frac{\sum_{i}{\left(x_{i}-y_{i}\right)}^{2}}{N}} \end{array}$$

where *N* represents the total number of measurements; *x*_*i*_ is the manual counting; *y*_*i*_ is the automatic counting, and ȳ is the mean.

### Code and Data Availability

The detection and segmentation models were all developed using the PyTorch software library (Facebook Artificial Intelligence Research Institute, FAIR), which is an open-source Python deep learning library. The code is fully open-source for academic usage and can be downloaded at https://github.com/WeizhenLiuBioinform/stomatal_index. The wheat dataset is available for downloading at https://github.com/WeizhenLiuBioinform/stomatal_index/releases/download/wheat1.0/wheat_dataset.zip.

## Results and Discussion

The hardware for training the proposed stomatal index measurement pipeline is a GPU server equipped with an Intel Xeon(R) E5-2650 CPU and four GeForce GTX 1080Ti GPUs with 11G memory, but only two of the four GPUs were used. The pipeline was implemented using the PyTorch framework running on the CentOS 7.7 operating system.

### Stomata Detection

The Faster R-CNN-based stomata detection model was set up with the initial learning rate of 0.0005 and the batch size of 16. It was trained over 20 epochs. To evaluate the stability and reliability of the model, we conducted five-fold cross-validation that shifted the training and test sets for each fold. The 1,000 microscopic images of leaf epidermis were randomly divided into five mutually exclusive subsets. One subset was used as the validation set (200 images), and the other four were used as the training set (800 images). Offline data augmentation was performed to expand each subset by applying rotations of 45, 90, and 135°, respectively, to each image and keeping the original images. By these geometric transformations, 4,000 images were obtained that can be used to enhance the robustness of the model to different stomata angles.

**Figure 4:**
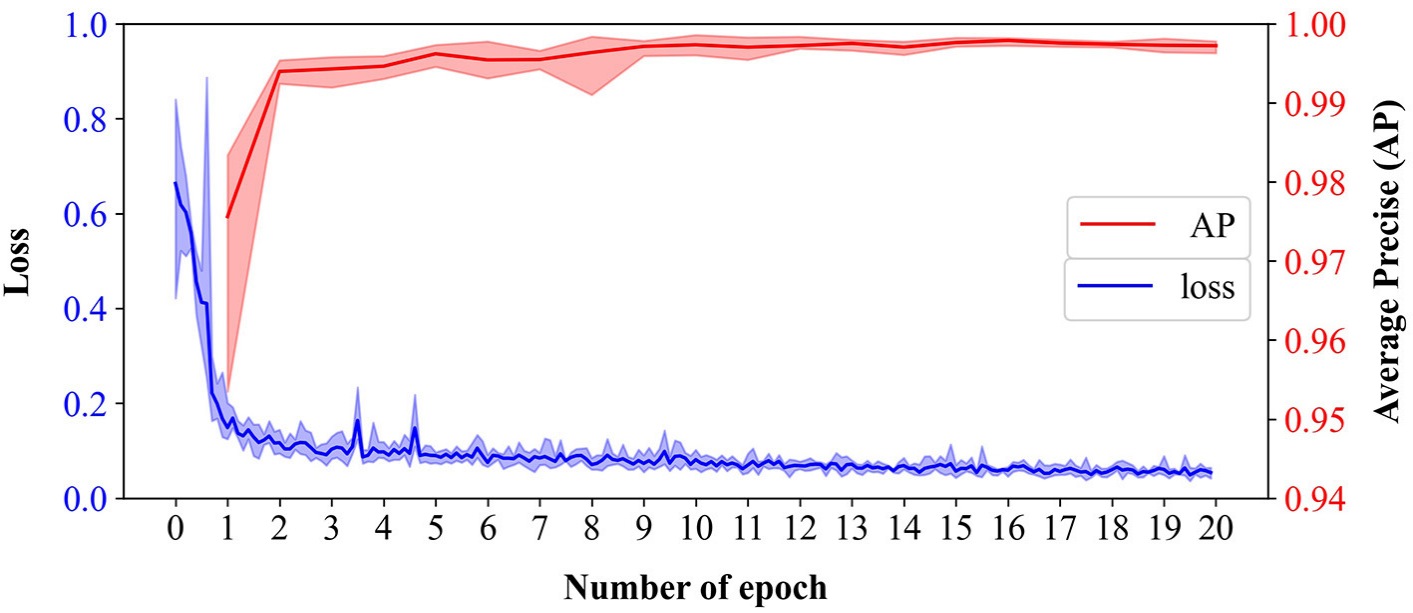
The evolutionary curves of loss of training sets and average precision (AP) of validation sets of the Faster R-CNN-based model across 20 epochs from five-fold cross-validation. Bold lines represent the mean of loss (blue) and AP (red), and the translucent bands represent the range of loss and AP across five-fold.

The stomata detection results of the proposed model were quite satisfactory, which achieved a mean validation AP of 0.997 across the five-fold cross-validation with an SD = 0.000521. The evolutionary curves of the AP and loss over 20 epochs are shown in Figure 4. An “epoch” was defined as the process of training the model once using all of the images in the training set. In this study, we took the prediction bbox with the intersection over union (IoU) of ground truth >0.6 as the true positive. The curves showed a good learning ability since the loss of training sets decreased rapidly in the first two epochs and reached a small value of 0.12 after five epochs. The AP of validation sets rose rapidly that reached 0.975 after the first epoch. It had satisfactory convergence after 10 epochs until finally reaching its optimal prediction performance. The fast convergence is also due to adopting the ResNet101 pretraining weight on ImageNet to initialize the feature extraction network. All the AP curves of the five-fold cross-validation were close to each other with small fluctuations before the first 10 epochs, illustrating the high stability and reliability of the model for stomata detection.

**Figure 5:**
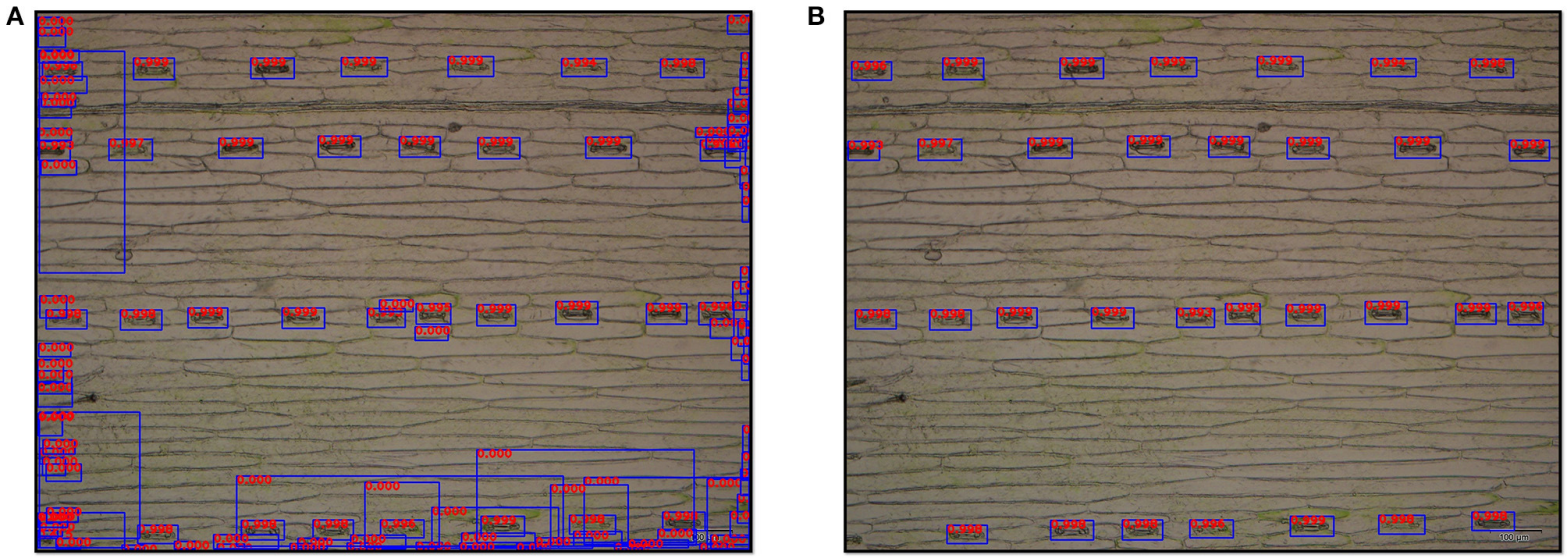
Output images of stomata detection results by the Faster R-CNN-based model at ×10 magnification. **(A)** Original detection result with all proposal bboxes (112 bboxes), and the number on the bbox represents its detection score. **(B)** Detection result after filtering (32 stomata).

An example of the stomata detection result conducted was present in Figure 5. The Faster R-CNN-based model generated 112 proposal bboxes (Figure 5A). After applying the filtration with a confidence threshold value of 0.9, all 32 stomata on the leaf epidermal image were accurately detected (Figure 5B).

### Epidermal Cell Segmentation

The UNet-based epidermal cell segmentation model was set up with a batch size of 8 and an initial learning rate of 0.0001. Unlike the Faster R-CNN-based stomata detection model, it was trained over 200 epochs, since it decayed to 0.00001 by the cosine annealing algorithm until 200 epochs. We also performed the five-fold cross-validation using the same 1,000 microscopic images as stomata detection. Online augmentation was used to enlarge the image dataset. This augmentation focuses on “batches,” which refers to various transformations of images during training to increase the diversity of image samples. The number of iterations can be increased to ensure that the number of images for training increases. In this study, in each batch, before the data being fed into U-Net, online data augmentation was performed to transform images and the corresponding ground truths by applying affine transformation with a probability of 0.2 and rotating 90° with a probability of 0.5.

**Figure 6:**
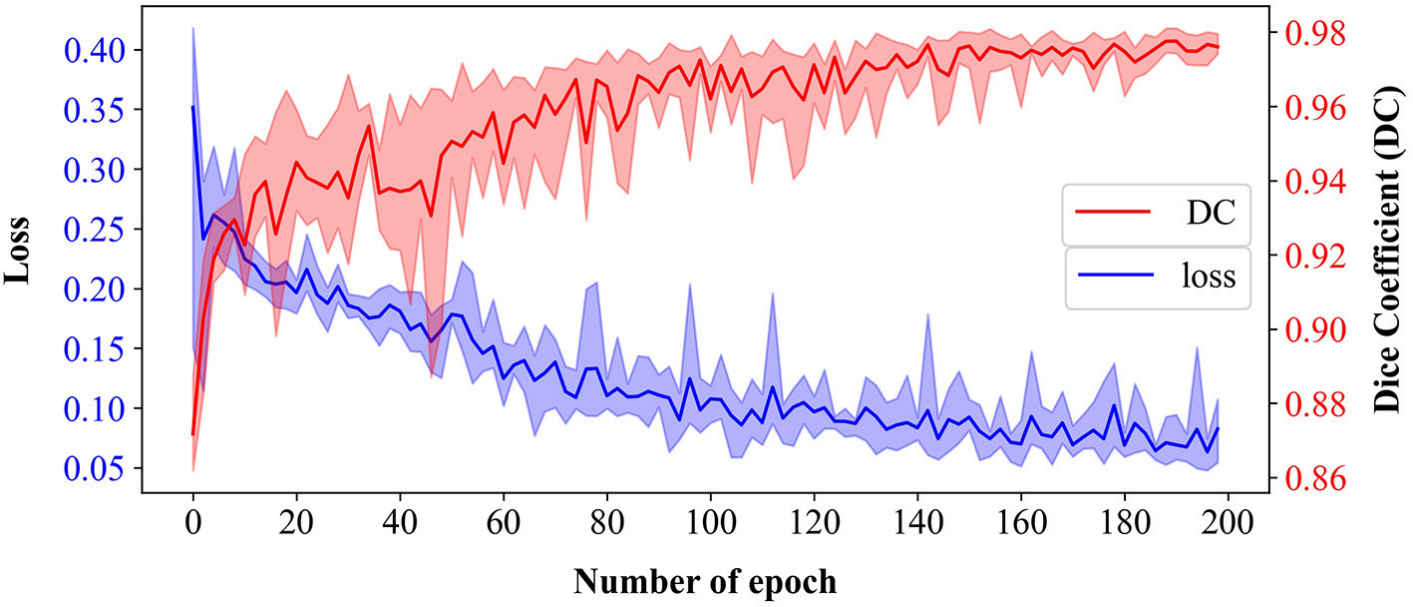
Training loss and validation dice coefficient (DC) of U-Net-based model over 200 epochs from five-fold cross-validation. Bold lines represent the mean of loss (blue) and DC (red), and the translucent bands represent the range of loss and DC across five-fold.

The epidermal cell segmentation of the proposed model achieved the mean DC of 0.978 with SD = 0.00121 across the five-fold cross-validation that demonstrates segmentation performances of the models which are reliable and stable. As shown in Figure 6, the U-Net model started to converge after 100 epochs. Although the loss of the training sets fluctuated, the DC stabilized above 0.97 after 150 epochs and finally reached its optimal performance over 200 epochs. During the whole convergence, the DC fluctuated upward, while the loss continued in a fluctuational decline, indicating that the models were continuously learning rather than being trapped in a local optimal.

**Figure 7:**
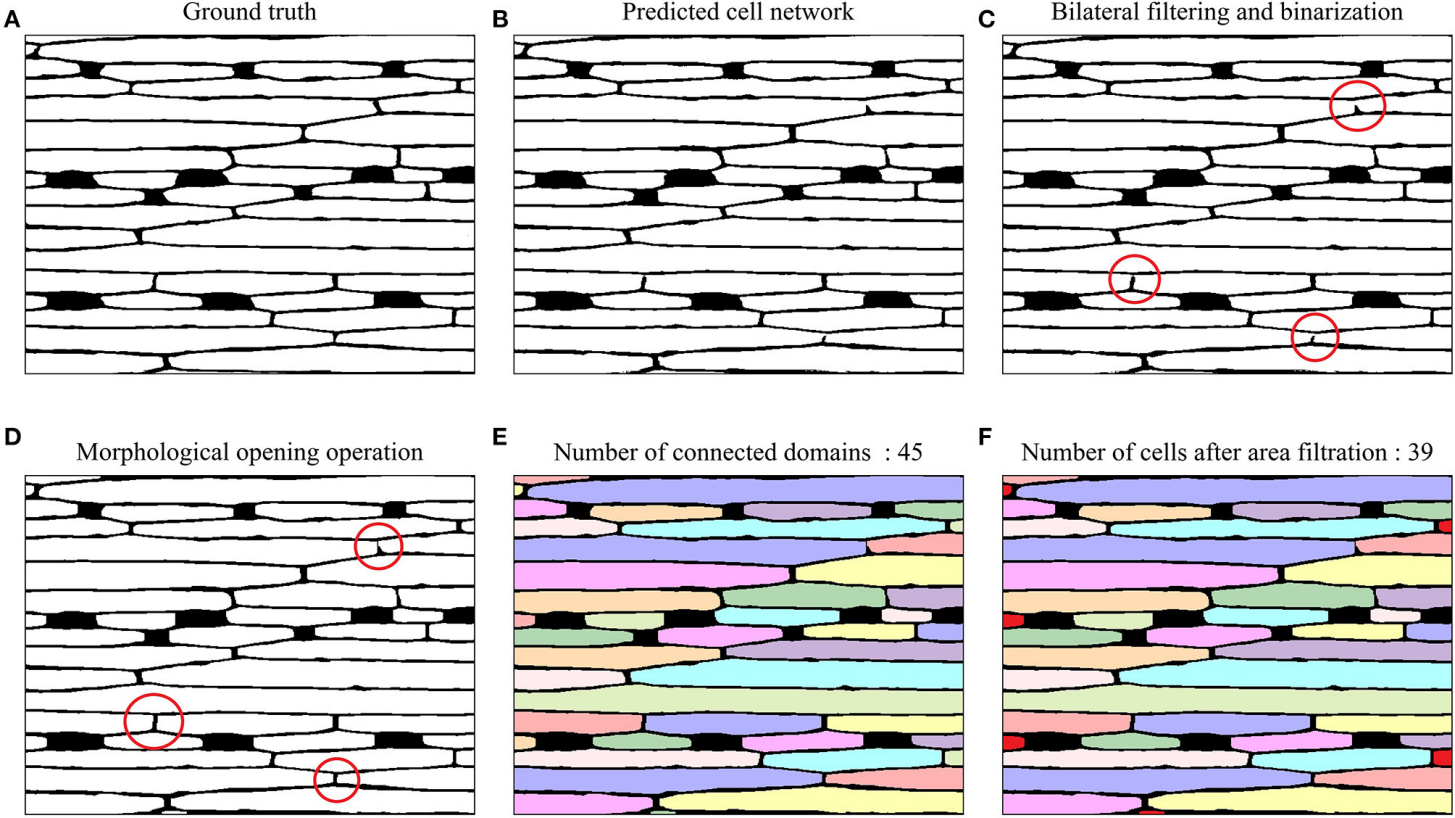
Example images of epidermal cell segmentation by the U-Net-based model and morphological post-processing on a 20× magnified microscope image of the wheat leaf. **(A)** Ground truth. **(B)** Epidermal cell network predicted by U-Net. **(C)** Bilateral filtering and binarization; Red circles highlighted the breakpoints in the cell network. **(D)** Morphological opening operation; red circles highlighted connected breakpoints in the cell network after the morphological opening operation. **(E)** Connected domains with different colors (45 connected domains). **(F)** Epidermal cell counting after area filtering (39 epidermal cells). The six small connected domains that were filtered out were marked with red color.

For epidermal cell segmentation, the U-Net-based model predicted the epidermal cell network (Figure 7). Comparing to the ground truth (Figure 7A), the predicted cell network (Figure 7B) had several breakpoints in some cell walls, which affected the cell counting accuracy because the connected domain method was used for counting. To connect these breakpoints, a series of image-processing techniques were utilized, including bilateral filtering, binarization, and morphological opening operation (Figures 7C,D). Considering the incomplete cells present in the image, before cell counting, the small connected domains were filtered out whose areas were <1/10 of the mean area of connected domains. In Figures 7E,F, after this area filtering, 39 connected domains remained as the epidermal cells, while six small connected domains at the edge (filled with red) were not counted.

### Performance Evaluations of Stomatal Index Measurement

#### Accuracy and Precision

We counted the number of stomata and epidermal cells for all the microscopic images over the five-fold cross-validation using the proposed Faster-RCNN and U-Net-based models, respectively. For each fold, their counting accuracies were estimated and summarized in Table 1. Both models had good counting accuracy, ranging from 97.577 to 98.451% for stomata and 94.584 to 95.330% for epidermal cells. The epidermal cell model had slightly lower counting accuracy than the stomata model. This result was easily understood because morphological features of epidermal cells were less distinct from the background than stomata, making accurately count the numbers of epidermal cells more challenging. Since the high-counting accuracies and precisions of stomata and epidermal cells, as expected, the stomatal index, which is the ratio of stomata number over the total numbers of stomata and epidermal cells, also achieved a high accuracy of 95.35%.

**Table 1:** The counting accuracies of stomata and epidermal cells over 5-fold cross-validation.

**Trait**	**Accuracy (%)**					
	**Fold_1**	**Fold_2**	**Fold_3**	**Fold_4**	**Fold_5**	**Mean**
Stomata	97.675	98.058	97.577	98.451	98.397	98.031
Epidermal cell	94.584	95.106	95.33	94.977	95.168	95.033

**Figure 8:**
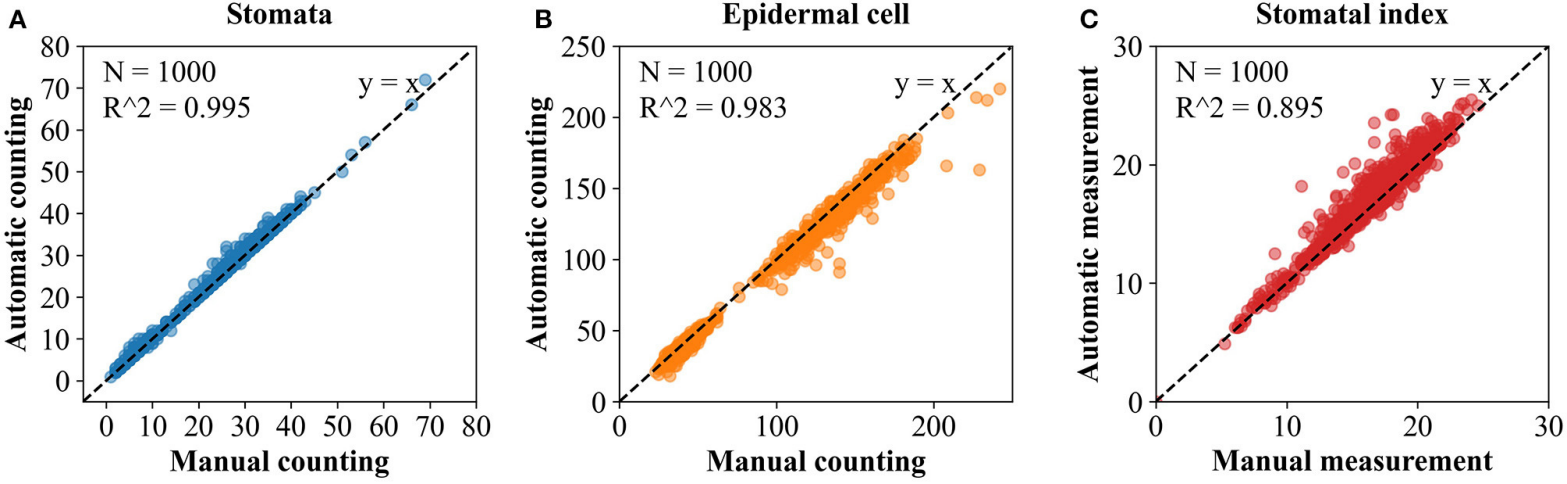
Simple linear regression between the manual and automatic measurement. **(A)** Stomata. **(B)** Epidermal cell. **(C)** Stomatal index.

To further evaluate the differences between results of automatic counting by the proposed pipeline and ground truths, the numbers of stomata and epidermal cells were counted manually for all the 1,000 microscopic images in the dataset. Regression analysis was performed between the manual and automatic counting (Figure 8). *R*^2^ for stomata, epidermal cells, and stomatal indices were 0.995, 0.983, and 0.895, respectively, and the RMSE values were 0.821, 6.460, and 1.099, respectively. These results verified strong correlations between manual and automatic counting results. Counting and measurement precisions were also estimated to detect the overcounting (negative values) and undercounting (positive values) errors. As the distributions shown in Table 2, the means of counting precisions for stomata and epidermal cells, and stomatal index were very close to 0 (−0.009, 0.019, and −0.016, respectively) with SD = 0.024, 0.027, and 0.023, respectively. Overall, automatic counting results in most of the microscopic images were identical to manual counting results, illustrating the high precision of the proposed pipeline. To be more specific, the Faster R-CNN-based stomata counting algorithm was a little bit more likely to overcounting, while the U-Net-based epidermal cell counting algorithm was more likely to undercounting. The stomatal index using the proposed pipeline was prone to be slightly overestimated.

**Table 2:** The precisions of stomata and epidermal cell counting and stomatal index measurement in 1,000 microscopical images of the wheat dataset.

**Trait**	**Precision**			
	**Min**	**Max**	**Mean**	**SD**
Stomata	−0.204	0.067	−0.009	0.024
Epidermal cell	−0.056	0.250	0.019	0.027
Stomatal index	−0.214	0.097	−0.016	0.023

#### Average Running Time

We estimated the speed of the proposed automatic analysis pipeline for stomatal index measurement using the 1,080 Ti GPU and CPU. The ART of the pipeline mainly came from the counting time for stomata and epidermal cells and the calculation time of the stomatal index (Formula 1). From inputting the microscopic image to outputting the stomatal index value, the ART per image was only 0.32 s using the GPU *via* the matrix acceleration calculation but 7.49 s on the CPU. Specifically, the ART for the stomata counting was 0.15 and 5.96 s using GPU and CPU, respectively. As the ART for the stomata counting part was comprised by the time of inference of Faster R-CNN-based stomata detection model and non-maximum suppression (NMS) of bboxes, the large difference of ART using GPU and CPU came mainly from the NMS process. For the epidermal cell counting, the ART, including the time of inference of the cell segmentation model and the morphological postprocessing time, was 0.17 and 1.53 s using the GPU and CPU, respectively. The speed difference came from the inference of the U-Net-based-segmentation model running on the different hardware because the post-processing procedures could only be run on the CPU. Overall, the proposed automatic pipeline for stomatal index measurement had an excellent running efficiency.

#### Stomatal Index Values at Different Magnifications

Using the proposed pipeline, we explored the measurement results of stomata number, epidermal cell number, and stomatal index of wheat leaf epidermal at ×10 and ×20 magnifications (Table 3). A total of 50 brand new images that were not used to train the pipeline at ×10 magnification were selected and used to generate 50 images at ×20 magnification by cropping and reshaping. The mean numbers of stomata and epidermal cells at ×10 magnification were 27.16 and 133.82, which were more than four times compared with those at ×20 magnification. More importantly, the means of the stomatal indices at ×10 and ×20 magnifications were 16.76 and 13.70, which were significantly different from each other (pairwise *T*-test: *t* = 5.752, *df* = 49, and *P* = 5.623E-7). The same trend was also observed in the 1,000 images (500 images at ×10 and ×20 magnifications, respectively) previously used for training the pipeline (Supplementary Table 3). This systematic difference hints that the larger field of view, the higher the stomatal index value from the same leaf. When comparing values of the stomatal index among different samples or genotypes, we should make sure they are at the same magnification.

**Table 3:** Summary of average numbers of stomata and epidermal cells and the stomatal index trait in 50 epidermis images at × 10 and × 20 magnifications, respectively.

**Magnification**	**Stomata**			**Epidermal cell**			**Stomatal index (%)**		
	**Min**	**Max**	**Mean**	**Min**	**Max**	**Mean**	**Min**	**Max**	**Mean**
10x	14	53	27.16	83	209	133.82	10.83	20.99	16.76
20x	0	17	6.44	24	60	38.66	0	24.29	13.70

#### Comparison Between Stomatal Index and Stomatal Density

The stomatal index and stomatal density of two wheat varieties were assessed (Gharflor-1611 and Ningmai9) using the proposed pipeline. Stomatal density was defined as the number of stomata divided by the area of the field of view. Five micrographics at ×10 magnification were sampled for each variety, and the field of view of each image is 1.428 mm^2^. As expected, a smaller coefficient of variation was obtained for the stomatal index than the stomatal density in two wheat cultivars (Table 4). It illustrates that the stomatal indices were more constant than stomatal densities within a cultivar. Moreover, Pearson's correlation coefficients between stomatal density and stomatal index were high, which are 0.878 and 0.926 for Gharflor-1611 and Ningmai9, respectively.

**Table 4:** Summary of the stomatal index and stomatal density characters of two wheat varieties.

**Cultivar**	**Stomatal density (pores|mm** ^****2****^ **)**			**Stomatal index (%)**			**Correlation coefficient**
	**Range**	**Mean**	**CV**	**Range**	**Mean**	**CV**	
Gharflor-1611	17.503–26.605	21.704	0.134	16.779–19.388	17.922	0.051	0.878
Ningmai9	2.800–9.802	7.000	0.329	3.810–9.910	8.066	0.271	0.926

*CV, coefficient of variation; correlation coefficient of each cultivar was calculated using stomatal density values and stomatal index values of five micrographs*.

### Applications on Other Plant Families

How well the proposed pipeline can be applied to other plant families is an interesting point worth studying. The cuticle dataset with 156 micrographs collected from 31 plant families (Supplementary Table 2) was used. Considering the morphological differences of stomata and epidermal cells between the wheat (Figure 1) and the cuticle dataset (Supplementary Figure 1), transfer learning was employed that the Faster RCNN and U-Net were initialized using the model parameters in the wheat dataset and all parts of the models were then finetuned by the training set of the cuticle dataset. In this way, an excellent model can be trained using a small amount of data (Zhuang et al., 2020). To avoid the possible overfitting of the model parameters to the wheat dataset, the intermediate checkpoints were selected (the 10th epoch of Faster RCNN and the 100th epoch of U-Net) in the training process of the wheat dataset as the pretraining model and finetuned the model with a smaller initial learning rate (0.0001 for Faster RCNN and 0.00005 for U-Net). A total of 105 images in the cuticle dataset were used as the training set and 51 images as the testing set (Supplementary Table 2). After 20 epochs for Faster RCNN and 100 epochs for U-Net, the models reached convergences.

**Figure 9:**
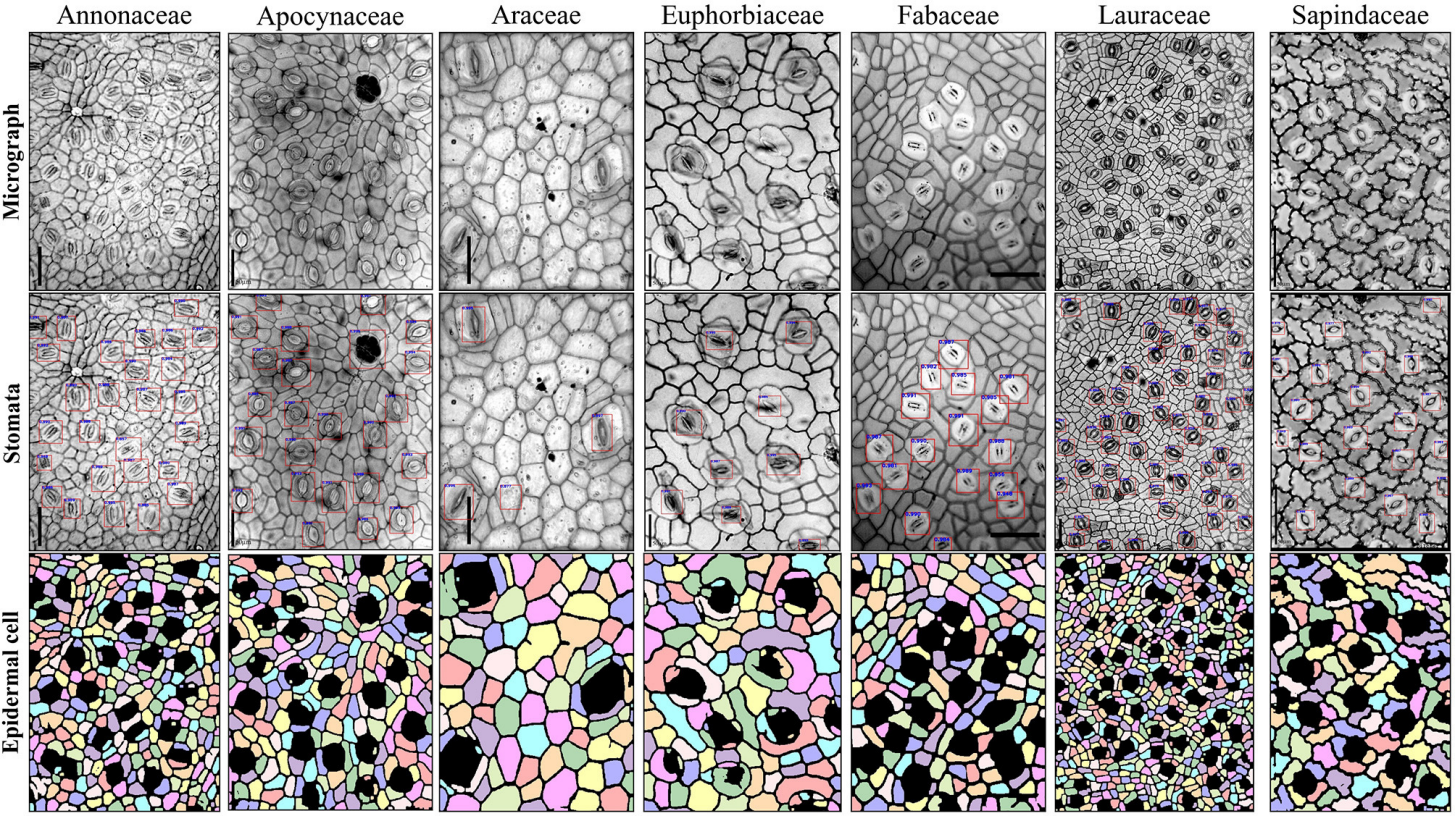
Example images of seven plant families in the testing set of cuticle dataset using stomata detection and epidermal cell segmentation algorithms.

The finetuned pipeline achieved good counting accuracies and precisions for stomata and epidermal cells on the testing set derived from seven plant families (Table 5 and Figure 9). The average counting accuracies of all families were 94.355% for stomata and 91.127% for epidermal cells, and the stomatal index accuracy reached 89.384%. The counting precisions for stomata, epidermal cells, and stomatal index were very close to 0 (0.006, −0.02, and 0.023, respectively). In five of seven families, the Faster RCNN-based stomata counting model achieved better performance than the U-Net-based epidermal cell counting model. The same situation was observed in the wheat dataset. In the Araceae family, all the stomata were accurately predicted, and the counting accuracy of epidermal cells reached 93.895%. Therefore, the stomatal index accuracy (94.63%) was the highest in all the families. The Euphorbiaceae family obtained the lowest counting accuracy of the stomatal index (82.68%) due to the relatively low-counting accuracy of epidermal cells.

**Table 5:** Summary of performance evaluations of the proposed pipeline on the cuticle dataset.

**Plant family**	***N*_**image**_**	**Stomata**				**Epidermal cell**				**Stomatal index**			
		**CA (%)**	**CP**	***R*^**2**^**	**RMSE**	**CA (%)**	**CP**	***R*^**2**^**	**RMSE**	**SIA(%)**	**SIP**	***R*^**2**^**	**RMSE**
Annonaceae	6	92.59	0.034	0.89	2.68	94.58	0.02	0.97	10.71	90.11	0.046	0.71	1.9
Apocynaceae	7	92.49	−0.001	0.96	1.41	90.65	−0.001	0.94	12.83	85.55	−0.0002	0.65	2.56
Araceae	5	100	0	1	0	93.9	−0.02	0.96	9.45	94.63	0.019	0.95	0.43
Euphorbiaceae	7	93.36	0.032	0.98	1	82.35	−0.064	0.58	18.81	82.68	0.084	0.43	2.61
Fabaceae	7	91.19	−0.017	0.88	1.46	95.18	0.002	0.98	5.2	89.22	−0.067	0.96	1.12
Lauraceae	13	94.85	−0.0003	0.98	1.98	91.46	−0.001	0.97	19.72	92.19	0.008	0.91	0.92
Sapindaceae	6	97.37	0.012	0.98	0.91	90.72	−0.027	0.71	16.98	90.68	0.034	0.67	1.42
All	51	94.36	0.006	0.98	1.63	91.13	−0.02	0.97	15.17	89.38	0.023	0.84	1.7

*N_image_, the number of microscopic images used for individual plant family; CA, counting accuracy; CP, counting precision; SIA, stomatal index accuracy; SIP, stomatal index precision; R^2^ and RMSE were obtained from the simple linear regression (y = x) between manual counting by experts and automatic counting*.

Regression analysis on the cuticle test set was performed between the manual and automatic counting (Table 5 and Supplementary Figure 2). *R*^2^ for stomata, epidermal cells, and the stomatal index was 0.976, 0.967, and 0.841, respectively, and the RMSE values were 1.627, 15.17, and 1.704, respectively, indicating that the proposed pipeline also showed excellent performances in many other species besides wheat.

## Conclusions and Future Work

In this study, an automatic deep learning-based method was proposed for measuring stomatal index, taking microscope images of wheat leaves as the input. The proposed method consisted of three parts that were the Faster R-CNN target detection algorithm for detecting and counting the stomata; the U-Net semantic segmentation network for extracting the epidermal cell network and measuring the number of connected domains as the number of epidermal cells; and subsequently calculated stomatal index of each image using the previous counting results. Satisfactory accuracies were obtained for stomata detection and counting, cell segmentation and counting, and stomatal index measurements. High correlations were observed between manual and automatic methods. In addition, the proposed image analysis pipeline was quite fast. Using the GPU (1080Ti), it took only 0.32 s to estimate the stomatal index of an image. It should be noticed that a possible difference in the stomatal index could be identified from the same leaf at different magnifications. If using this trait as a diagnostic characteristic for a given genotype or species, magnification of the microscopic images should be taken into consideration. The wheat stomatal index assessment model also exhibited a promising transferability on the other plant species. Using a small number of images to finetune the model, it achieved good accuracies and precisions on seven plant families.

The proposed pipeline regarded stomata and epidermal cell counting as two independent tasks and trained their neural networks separately. In reality, they are related to each other. Multitask deep neural network, as a subfield of machine learning, solves multiple tasks simultaneously by taking advantage of the sharing representations between related tasks. It was utilized successfully across many applications in computer vision (Zhang et al., 2014; Li et al., 2016). In the future, we can attempt the multitask deep neural network to improve the performance of the proposed stomatal index measurement pipeline. The hidden layers of the stomatal detection and epidermal cell segmentation networks can be soft- or hard-shared to obtain an end-to-end model for stomatal index estimation, possibly achieving a better generalization ability and a faster analysis speed.

## Data Availability Statement

The datasets presented in this study can be found in online repositories. The names of the repository/repositories and accession number(s) can be found in the article/Supplementary Material.

## Author Contributions

WL, XY, and CZhu conceived the study. WL and CZhu wrote the manuscript. HM, SL, and FL collected the microscopic images of the leaf epidermis. CZhu, YH, CZha, and LL annotated the datasets. CZhu, YH, WL, and XY designed the stomatal index measurement pipeline. CZhu wrote PYTHON scripts. All authors edited and approved the manuscript.

## Conflict of Interest

The authors declare that the research was conducted in the absence of any commercial or financial relationships that could be construed as a potential conflict of interest.

## Publisher's Note

All claims expressed in this article are solely those of the authors and do not necessarily represent those of their affiliated organizations, or those of the publisher, the editors and the reviewers. Any product that may be evaluated in this article, or claim that may be made by its manufacturer, is not guaranteed or endorsed by the publisher.

## Supplementary Material

Supplementary Figure 1 Microscopic images of the cuticle dataset. **(A)** Training set. **(B)** Testing set.

Supplementary Figure 2 Simple linear regression between the manual and automatic measurement in the cuticle dataset. **(A)** Stomata. **(B)** Epidermal cell. **(C)** Stomatal index.

Supplementary Table 1 Variety information of wheat (*Triticum aestivum* L.) dataset.

Supplementary Table 2 Description of the cuticle dataset used for training and testing the stomatal index measurement model.

Supplementary Table 3 Summary of average numbers of stomata and epidermal cells and the stomatal index trait in 500 epidermis images at ×10 and ×20 magnifications, respectively, in the wheat dataset.
