# Supplementary material for: A Deep Learning-Based Method for Automatic Assessment of Stomatal Index in Wheat Microscopic Images of Leaf Epidermis
Source: Front Plant Sci. 2021 Sep 3;12:716784. doi: 10.3389/fpls.2021.716784 (PMC8446633; doi:10.3389/fpls.2021.716784)
Supplement: Supplementary Figure 1 — Microscopic images of the cuticle dataset. (A) Training set. (B) Testing set. [file Data_Sheet_1.zip › Supplementary Table S1.DOCX]

**Table S1.** Variety information of wheat (*Triticum aestivum* L.) dataset.

| Lumai11 | Qingfeng1 | Yannong24 | Emai17 |
| --- | --- | --- | --- |
| Ningmai13 | Yumai18(Aizao781) | huaimai20 | Ningchun13 |
| Nongda36 | Nongda1108 | Zhongmai175 | Weimai4 |
| Shannong1 | Yangmai12 | Jimai26 | Fielder |
| Wanmai50 | VAIOLET | Xinong88 | Emai6 |
| Xiaoyan4 | Xinmai26 | Shannong15 | Wanmai31 |
| Xiaoyan759 | Yangmai13 | Jinmai31 | Fengkang8 |
| Xuzhou8 | Jiaomai266 | Emai18 | Bima4 |
| Yumai16 | Wanmai33 | Emai12 | Beijing10 |
| Yumai19 | Yannong22 | Wanmai19 | Longchun8 |
| Chuanmai42 | Yunong949 | Shannong12 | Sumai3 |
| Zhengzhou5 | Xingmai1 | Jinmai47 | Beijing8 |
| Zhoumai28 | Xiaoyan22 | Chuanmai8 | GHARFLOR-1611 |
| Zhoumai32 | Linmai4 | Emai15 | Zhoumai26 |
| Qinmai1 | Zhengmai379 | Ningmai15 | Fengchan1 |
| Xiannong39 | Zhoumai23 | Yangmai17 | Jimai19 |
| Yangmai2 | Shanmai150 | Yangmai1 | Jimai20 |
| Zhoumai13 | Xi'an8 | Jinmai54 | Jimai22 |
| Zhoumai20 | Mianmai46 | Yangmai11 | Mianyang31 |
| Aikang58 | Zhengmai9023 | Emai16 | Yumai10 |
| Yumai49 | Sumai6 | Jimai1 | Yumai25 |
| Kenong199 | Gaoyou503 | Zhemai1 | Xinong979 |
| Jimai22 | Zhengmai9201 | Mianmai40 | Dongfanghong3 |
| Ningmai9 | Zhongmai9 | Ningmai3 | Fengkang7 |
| Jimai20 | Yangmai16 | Fengchan3 | Jimai3 |
