# Supplementary material for: A Deep Learning-Based Method for Automatic Assessment of Stomatal Index in Wheat Microscopic Images of Leaf Epidermis
Source: Front Plant Sci. 2021 Sep 3;12:716784. doi: 10.3389/fpls.2021.716784 (PMC8446633; doi:10.3389/fpls.2021.716784)
Supplement: Supplementary Figure 1 — Microscopic images of the cuticle dataset. (A) Training set. (B) Testing set. [file Data_Sheet_1.zip › Supplementary Table S2.DOCX]

**TABLE** **S2**. Description of the cuticle dataset used for training and testing the stomatal index measurement model.

| **Training set (30 plant families, 105 images)** | | | | | |
| --- | --- | --- | --- | --- | --- |
| **Plant family** | ***N*_images_** | **Plant family** | ***N*_images_** | **Plant family** | ***N*_images_** |
| Anacardiaceae | 1 | Fabaceae | 2 | Pircfamnia | 2 |
| Annonaceae | 2 | Fagaceae | 3 | Proteaceae | 4 |
| Apocynaceae | 6 | Lauraceae | 13 | Rubiaceae | 1 |
| Araceae | 9 | Magnoliacea | 1 | Santalaceae | 3 |
| Araliaceae | 2 | Meliaceae | 2 | Sapindaceae | 10 |
| Arecaceae | 11 | Monimiaceae | 2 | Simaroubaceae | 1 |
| Caryocaraceae | 1 | Myristicaceae | 1 | Taxaceae | 1 |
| Chrysobalanaceae | 2 | Myrsinaceae | 5 | Theaceae | 2 |
| Cyperaceae | 1 | Myrtaceae | 1 | Typhaceae | 1 |
| Dipterocarpaceae | 1 | Palmae | 12 | Winteraceae | 2 |
| **Testing set (7 plant families, 51 images)** | | | | | |
| Annonaceae | 6 | Euphorbiaceae | 7 | Sapindaceae | 6 |
| Apocynaceae | 7 | Fabaceae | 7 |  |  |
| Araceae | 5 | Lauraceae | 13 |  |  |
