# Supplementary material for: A Deep Learning-Based Method for Automatic Assessment of Stomatal Index in Wheat Microscopic Images of Leaf Epidermis
Source: Front Plant Sci. 2021 Sep 3;12:716784. doi: 10.3389/fpls.2021.716784 (PMC8446633; doi:10.3389/fpls.2021.716784)
Supplement: Supplementary Figure 1 — Microscopic images of the cuticle dataset. (A) Training set. (B) Testing set. [file Data_Sheet_1.zip › Supplementary Table S3.DOCX]

**TABLE S3.** Summary of average numbers of stomata and epidermal cells and the stomatal index trait in 500 epidermis images at 10x and 20x magnifications, respectively, in the wheat dataset.

|  | Stomata | | |  | Cell | | |  | Stomatal index (%) | | |
| --- | --- | --- | --- | --- | --- | --- | --- | --- | --- | --- | --- |
|  | min | mean | max |  | min | mean | max |  | min | mean | max |
| 10x | 4 | 29.13 | 71 |  | 60 | 131.53 | 220 |  | 6.25 | 18.06 | 25.18 |
| 20x | 0 | 7.44 | 16 |  | 18 | 39.15 | 66 |  | 0 | 15.86 | 25.49 |
