# Supplementary figures and images for: A Deep Learning-Based Method for Automatic Assessment of Stomatal Index in Wheat Microscopic Images of Leaf Epidermis
Source: Front Plant Sci. 2021 Sep 3;12:716784. doi: 10.3389/fpls.2021.716784 (PMC8446633; doi:10.3389/fpls.2021.716784)

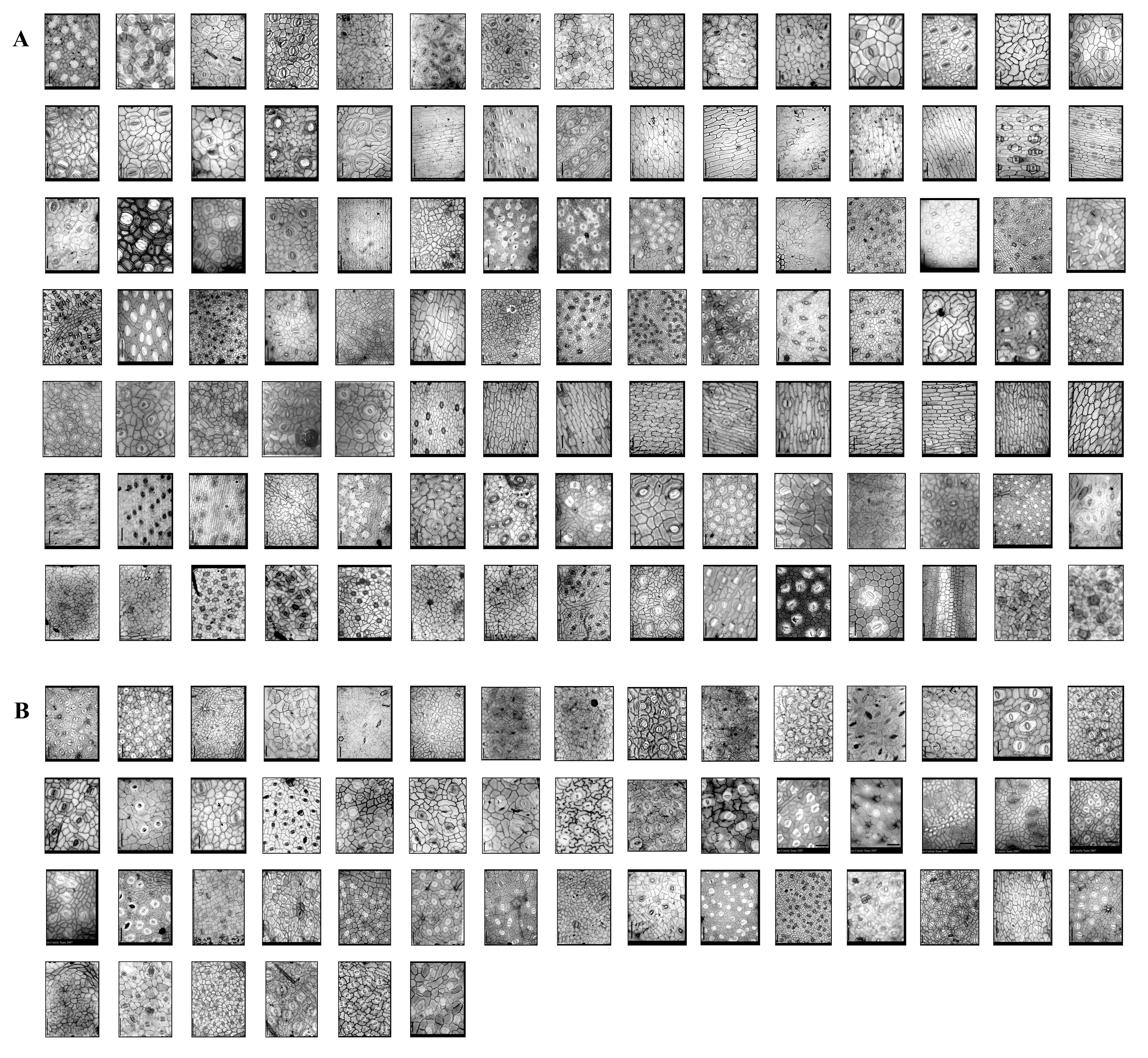

Supplement: Supplementary Figure 1 — Microscopic images of the cuticle dataset. (A) Training set. (B) Testing set. [file Data_Sheet_1.zip › Supplementary Figure S1.JPEG]

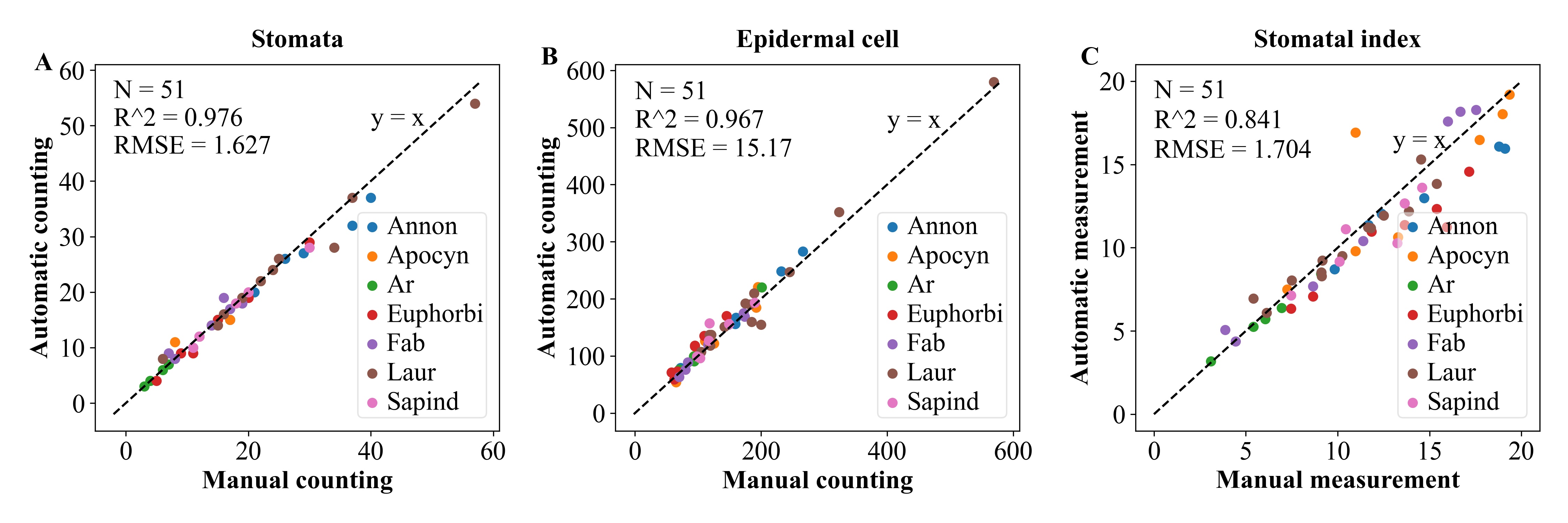

Supplement: Supplementary Figure 1 — Microscopic images of the cuticle dataset. (A) Training set. (B) Testing set. [file Data_Sheet_1.zip › Supplementary Figure S2.JPEG]
